# Evaluation of respiratory virus transmissibility and resilience from fomites: the case of 11 SARS-CoV-2 clinical isolates

**DOI:** 10.1128/aem.00774-25

**Published:** 2025-08-27

**Authors:** Sofia Sisti, Elena Criscuolo, Benedetta Giuliani, Mattia Cavallaro, Michela Sampaolo, Matteo Castelli, Roberto Burioni, Massimo Locatelli, Nicola Clementi

**Affiliations:** 1 Laboratory of Microbiology and Virology, Vita-Salute San Raffaele University 18985, Milan, Italy; 2 IRCCS San Raffaele Scientific Institute 9372, Milan, Italy; University of Nebraska-Lincoln, Lincoln, Nebraska, USA

**Keywords:** natural compounds, disinfection, persistence, influenza virus, SARS-CoV-2

## Abstract

The emergence of severe acute respiratory syndrome coronavirus 2 (SARS-CoV-2) caused a global pandemic, significantly impacting social and economic aspects of society. While belonging to the *Coronaviridae* family, SARS-CoV-2 exhibited unique characteristics that facilitated its widespread transmission and pathogenicity. This study aimed to investigate the molecular characterization and infection kinetics of emerging SARS-CoV-2 variants, focusing on four key virological aspects: viral entry mechanisms, replication dynamics, immune evasion strategies, and surface persistence. We isolated 11 SARS-CoV-2 variants from clinical samples and conducted *in vitro* studies using Calu-3 cell lines and pseudovirus systems to explore viral entry. Whole-genome sequencing identified mutations linked to increased replication and enhanced immune evasion, particularly through the inhibition of interferon responses. Additionally, persistence studies on common environmental surfaces (copper, aluminum, and plastic) revealed that certain variants, such as G446S, contributed to increased viral stability, suggesting potential for indirect transmission beyond direct person-to-person contact. Furthermore, we evaluated the effectiveness of “green” disinfectants, including tea tree oil and quercetin-based compounds, which demonstrated viral load reductions of over 95% within minutes, outperforming conventional alcohol- and UV-based disinfection methods. Notably, two influenza virus isolates belonging to highly divergent subtypes are sanitized as well while showing different persistence surface profiles compared to SARS-CoV-2. These findings highlight the evolutionary strategies employed by SARS-CoV-2 to ensure transmission, its ability to adapt under selective pressure, and the importance of ongoing surveillance of emerging variants. Moreover, the efficacy of environmentally sustainable disinfectants presents a promising strategy for reducing viral transmission in both clinical and community settings.

**IMPORTANCE:**

In this study, we evaluated the molecular profile of 11 SARS-CoV-2 variants, focusing on four key virological aspects: viral entry mechanisms, replication dynamics, immune evasion strategies, and surface persistence. Through whole-genome sequencing, we identified mutations linked to enhanced replication and immune evasion, notably through the suppression of interferon responses. Additionally, persistence studies on common environmental surfaces (copper, aluminum, and plastic) demonstrated that certain mutations, such as G446S, exhibited increased stability, suggesting a potential role for indirect transmission. This study underscores the need for continuous monitoring and the potential of eco-friendly disinfection approaches in controlling the spread of SARS-CoV-2 and possibly of other respiratory viruses.

## INTRODUCTION

Since severe acute respiratory syndrome coronavirus 2 (SARS-CoV-2) emerged in late 2019, its high transmissibility has posed significant global health challenges. Unlike other RNA viruses, SARS-CoV-2 has relatively low genomic variability due to a proofreading mechanism in replication. However, it has accumulated mutations over time, modifying transmissibility and pathogenicity of virus variants that have outcompeted the original strain and become globally dominant (1, 2).

Mutations, mainly substitutions and deletions in the Spike protein, have led to World Health Organization (WHO)-classified variants of concern (VOCs) with increased transmissibility, severity, or reduced treatment efficacy (3). In contrast to the earlier variants, Omicron lineage (BA.) marked a significant shift in the virus evolution; the first BA. variant was identified in late 2021 and was characterized by over 30 mutations specifically in the receptor-binding domain (RBD) and N-terminal domain (NTD) of the S protein, leading to a remarkable ability to evade neutralizing antibodies, reducing the effectiveness of existing vaccines and prior immunity from earlier strains (4, 5). These changes in the viral S protein suggest a critical shift between pre-Omicron and Omicron variants, highlighting the virus’s adaptation to the human host and underscoring the need for continuous surveillance to address emerging variants (1). Mutations and their contribution to viral stability may enhance SARS-CoV-2 persistence on surfaces, potentially contributing to fomite transmission, although this mechanism remains less well understood. While primarily spreading via respiratory droplets, the virus can survive on surfaces for hours to days, influenced by factors like temperature, humidity, surface type, and viral load. Variants stability may be linked to mutations in structural proteins (S, M, and E). As a result, disinfection strategies are crucial in reducing surface transmission, especially in high-contact settings like healthcare facilities. Various disinfectants, including alcohol-based solutions and UV-C light, were tested for their efficacy against the first emerged SARS-CoV-2 variants, exhibiting effective virucidal activity (6, 7). Furthermore, some natural disinfectants, such as tea tree oil (TTO) and quercetin (QRE), and the biomolecule daptomycin (DAP), demonstrated promising antiviral activity against SARS-CoV-2 and other respiratory viruses (8, 9). While these disinfectants are critical for surface decontamination, the efficacy of each agent can be influenced by factors such as the surface type, viral variant, and environmental conditions.

This study explored the molecular characteristics and infection dynamics of emerging SARS-CoV-2 variants according to their mutation on virus-exposed proteins, focusing on main aspects related to virus transmission: viral entry, replication, immune evasion, and surface persistence.

## MATERIALS AND METHODS

### Virus sequencing

The viral RNA was extracted from 200 µL of heat-inactivated viral stock using the ELITe InGeniusTM workstation (ELITechGroup). Targeted RNA enrichment for Illumina sequencing was then performed using the BS-Paragon CleanPlex SARS-CoV-2 Flex Panel (Paragon Genomics) following the manufacturer’s instructions. The purified genetic material was quantified using the Qubit double-stranded DNA HS Assay Kit on a Qubit 2.0 Fluorometer (Thermo Fisher), and samples were diluted and pooled to a final concentration of 2 nM. Then, 50 pM of the pool was sequenced on the iSeq Illumina platform using the iSeq 100 i1 Reagent v.2 (300-cycle). The obtained data were then analyzed using the coronavirus antiviral and resistance database of Stanford University, through which the consensus sequence of the viral stock was obtained (https://covdb.stanford.edu/sierra/sars2/by-patterns). Then, the FASTA sequences were also verified through Nextclade software and deposited in the GISAID database (Global Initiative on Sharing All Influenza Data; https://gisaid.org) (Table 1).

**TABLE 1:** List of isolated variants of concern

Class	ID	Reference
Pre-Omicron virus	G614D	ID: EPI_ISL_413489
Pre-Omicron virus	Alpha	ID: EPI_ISL_1924880
Pre-Omicron virus	Beta	ID: EPI_ISL_1599180
Pre-Omicron virus	Gamma	ID: EPI_ISL_1925323
Pre-Omicron virus	Delta	ID: EPI_ISL_4198505
Omicron virus	BA.1	ID: EPI_ISL_12188061
Omicron virus	BA.2	ID: EPI_ISL_13285445
Omicron virus	BA.4	ID: EPI_ISL_13878328
Omicron virus	BA.5	ID: EPI_ISL_16355612
Omicron virus	BQ.1.1	ID: EPI_ISL_16355615
Omicron virus	XBB.1	ID: EPI_ISL_16526278
Omicron virus	XBB.1.5	ID: EPI_ISL_18537906
Omicron virus	EG.5	ID: EPI_ISL_18540450
Natural compound	TTO	Tea tree oil: W390208, Sigma-Aldrich
Natural compound	DAP	Daptomycin: SBR00014, Sigma-Aldrich
Natural compound	QRE	Quercetin: Q495, Sigma-Aldrich
Chemical compound	BFLA-1	Baflomycin A1: B1793, Sigma-Aldrich
Chemical compound	CAM	Camostat mesylate: SML0057, Sigma-Aldrich
Software	Nextclade v.3	https://docs.nextstrain.org/projects/nextclade
Software	MAFFT v.7	https://mafft.cbrc.jp/alignment/server/index.html
Software	MAESTROweb v.1.2.35	https://pbwww.services.came.sbg.ac.at/maestro/web

### Evaluation of kinetic profiles using real-time PCR

Calu-3 (3 × 10^5^ cells/mL) were seeded in 96-well plates and cultured for 3 days at 37°C and 5% CO_2_ in a humidified atmosphere. Then, the cells were infected with 0.001 multiplicity of infection (MOI) of variants isolated in triplicate. After 1 h of adsorption, the cells were washed three times with phosphate buffered saline (PBS) to remove any unbound virus, and cell supernatants were collected at 6 time points: 1, 3, 6, 24, 48, and 72 h post-infection (hpi). Viral genome from the collected supernatants was extracted with QIAamp Viral RNA Mini Kit (52904, QIAGEN). The Superscript III First-Strand system for RT-PCR (18080-051, Invitrogen) was used to synthesize cDNA from purified RNA following manufacturer’s instructions. Then, Real-Time PCR on the N gene was performed using SYBR Green PCR Master Mix (4309155, Applied Biosystems) and specific primers (Table 2). The thermal cycling conditions are the following: 95°C for 2 min, 45 cycles of 95°C for 20 s, 55°C for 20 s, 72°C for 30 s, and then 72°C for 10 min. Next, the replication kinetics of each variant over time were then summarized by calculating the area under the curve (AUC).

**TABLE 2:** List of primers and sequences

	Forward primer sequence	Reverse primer sequence
RdRp	CAAGTGGGGTAAGGCTAGACTTT	CTTAGGATAATCCCAACCCAT
IFN-β	TCCAAATTGCTCTCCTGTTG	GCAGTATTCAAGCCTCCCAT
IFIT1	TCTCAGAGGAGCCTGGCTAA	TGACATCTCAATTGCTCCAGA
MX1	CCAGCTGCTGCATCCCACCC	AGGGGCGCACCTTCTCCTCA
MX2	CAGAGGCAGCGGAATCGTAA	TGAAGCTCTAGCTCGGTGTTC
ISG15	CGCAGATCACCCAGAAGATCG	TTCGTCGCATTTGTCCACCA
RIG-I	ATCCCAGTGTATGAACAGCAG	GCCTGTAACTCTATACCCATGTC
GAPDH	TGGGCTACACTGAGCACCAG	GGGTGTCGCTGTTGAAGTCA
G446S	CAGCAAGCCGGGCGGCAACTACAAC	TCCAGCTTGTTGCTGTTC
N	TTACAAACATTGGCCGCAAA	GCGCGACATTCCGAAGAA

### Variant-specific immunoresponse in Calu-3 cells via real-time quantitative reverse transcription-PCR

Calu-3 cells (3 × 10^5^ cells/mL) were seeded into 96-well plates and cultured for 24 h at 37°C with 5% CO_2_ in a humidified atmosphere. Subsequently, the cells were infected in triplicate with 0.1 MOI of the G614D, Delta, Beta, BA.1, XBB.1, and XBB.1.5 isolated variants. After 1 h of adsorption, the cells were washed three times with PBS to remove any cell-free virus. Supernatants were then collected at six time points: 1, 3, 6, and 24 hpi. For RNA extraction, Calu-3 cells were lysed using RLT lysis buffer, and total cellular RNA was extracted using the RNeasy kit (74104, Invitrogen) according to the manufacturer’s instructions. Reverse transcription was performed using the First-Strand cDNA Synthesis System (18080-051, Invitrogen) with oligo(dT) primers to generate cDNA from the coding RNA. Quantitative RT-PCR analysis of interferon-stimulated gene (ISG) transcripts and human glyceraldehyde-3-phosphate dehydrogenase (GAPDH) was conducted using Power SYBR Green PCR Master Mix (4309155, Applied Biosystems, UK) with the following cycling conditions: initial denaturation at 95°C for 10 min, followed by 40 cycles of 95°C for 15 s and 60°C for 1 min. The sequences of the primers used are listed in the table below.

### Inhibition of pseudovirus entry process

To selectively inhibit different cell proteases involved in SARS-CoV-2 entry, two compounds were used: baflomycin A1 (BFLA-1) and camostat mesylate (CAM) (Table 1).

Calu-3 cells (3 × 10⁵ cells/mL) were seeded in 96-well plates and cultured for 3 days at 37°C with 5% CO_2_. Prior to pseudovirus transduction, cells were pretreated for 1 h with either BFLA-1 (100 nM), camostat mesylate (100 µM), or a combination of both. The pseudovirus transduction was performed using 100,000 relative luminescence units (RLU) for each variant, as determined by titration. A positive control, pseudovirus without inhibitors, was also included. Cells were centrifuged at 800 × *g* for 1 h at 37°C for adsorption and then incubated for 3 days at 37°C and 5% CO_2_. After incubation, cells were lysed with 100 µL of Glo Lysis Buffer (E2661, Promega) for 15 min at room temperature, and 100 µL of Bright-Glo Assay Reagent (E2620, Promega) was added just before luminescence detection using a Victor3 luminometer (Perkin Elmer).

Six biological replicates were performed for each condition. To evaluate the capability of the compounds to inhibit pseudoviral entry, transduction was normalized against uninfected cells (set as 100%) and untreated infected cells (set as 0%).

### Experimental protocol for viral persistence

#### SARS-CoV-2 variants

The persistence assay of 11 isolated SARS-CoV-2 variants was tested on three different materials—plastic, aluminum, and copper. Prior to use, all materials were thoroughly disinfected. We selected a reasonable viral load (1.5 × 10⁶ 50% tissue culture infectious dose [TCID_50_]/mL) that could be deposited on a surface by an infected individual, based on a previous study (6), and tested the same amount of the virus on each material on a 24-well plate. Following the inoculation, the virus was recovered from the surface by adding a volume of Dulbecco's modified Eagle medium (DMEM) (10 times the volume of the inoculum) after 15, 60, 120 min, and 6 h. The recovered virus was then aliquoted and stored at −80°C. To determine the viral persistence over time, the recovered virus was retitrated using a back-titration assay on Vero E6/TMPRSS2 (3 × 10^5^ cell/mL); after 1 h of adsorption, cells were washed with PBS to remove cell-free virus, and complete medium was added to cells supplemented with 2% fetal bovine serum (FBS). After 72 hpi, the cytopathic effect (CPE) was evaluated in inverted phase-contrast microscopy (Olympus CKX41), and TCID_50_/mL of persistent viruses was determined with the Reed-Muench formula. The infectious titer reduction rates were calculated as [1 – 1/10log10 (N0/Nt)] × 100 (%), where Nt is the titer of the post-persistence sample, and N0 is the titer of the sample before the persistence (10).

#### Influenza strains

The persistence of influenza A strains (A/Puerto Rico/8/34 H1N1 and A/Hong Kong/1/68 H3N2) was tested on plastic, aluminum, and copper using the same amount of virus (1.5 × 10^6^ TCID_50_/mL) in each material, following the same protocol applied to the SARS-CoV-2 variants to ensure consistency across experiments.

#### Pseudovirus XBB.1.5

Vero E6/TMPRSS2 cells (3 × 10⁵ cells/mL) were seeded in 96-well plates and incubated for 24 h. Serial 1:10 dilutions of pseudovirus were then added to the cells, followed by centrifugation at 800 × *g* for 1 h; after centrifugation, the media was removed, and the cells were washed with PBS. Fresh medium supplemented with 2% FBS was then added. At 72 hpi, the cells were lysed with 100 µL of Glo Lysis Buffer (E2661, Promega) for 15 min, and luminescence was measured by adding 100 µL of Bright-Glo Assay Reagent (E2620, Promega) and detecting signals using a Victor3 luminometer (Perkin Elmer). Based on these results, a standard of 100,000 RLU was selected for each pseudovirus to ensure equal viral input for subsequent persistence assays. Persistence was evaluated on sterilized plastic and aluminum surfaces at time points of 30 s, 1 min, 2 min, 5 min, and 30 min. To recover the pseudovirus, 10 times the volume of DMEM was added to the surface, and the samples were then stored at −80°C. After recovery, the pseudovirus was titrated back on Vero E6/TMPRSS2 cells, and persistence was quantified by measuring luciferase activity. For each variant, the persistence value was normalized to the stock titer (set as 100%).

### Disinfectant analysis protocol

To evaluate the disinfectant effectiveness of TTO, DAP, QRE, 95% ethanol (EtOH), and UV-C light, persistence experiments were repeated on sanitized plastic and aluminum surfaces using only the viral variants known for their prolonged stability. A total of 50 µL of viral variant titer was used, considering 1.5 × 10⁶ TCID_50_/mL. The experiments were performed within a 15 min window to ensure that the observed disinfectant effect could be attributed specifically to each disinfectant agent. For all disinfectants tested, the experiment was conducted in 12-well plates, where 50 µL of virus present on the surfaces was mixed with 50 µL of the disinfectant and recovered after 1, 5, and 10 min. For the UV-C treatment, used as a positive sanitation control, the samples were irradiated on ice at a dose of approximately 1.8 mW/cm², with a working distance of 20 cm for different exposure times (1, 5, and 10 min). After UV treatment, the virus was eluted, stored at −80°C, and subjected to back titration the following day, as described. After 72 h, the Vero E6/TMPRSS2 or MDCK cells were examined for CPE, and the TCID_50_/mL was calculated according to the previously described method.

### *In silico* characterization

#### Variant calling analysis

Whole-genome sequencing of the clinical isolates was performed, and the resulting sequences were aligned against the G614D reference variant. Variant calling analysis was then carried out on the S, M, and E proteins using the online MAFFT alignment program to identify potential mutations and variations.

#### Structural modeling and stability analysis of spike variants

The spike ectodomain (residues 14–1,146) was modeled using Modeller 10.4 in the 1-up and closed state, both as S0 and S1–S2 (processed at the furin cleavage site), using the spike sequence from the clinical isolate described in this study as query and the deposited structures with RCSB ID: GZGI and 7BNN as templates (11). To study the contribution of all the reported mutations of each variant, the last frame of each 200 ns trajectory was used for stability calculation using MAESTROweb (Table 1). More in detail, we evaluated stability in terms of free energy changes (ΔΔGbinding = ΔGmutant − ΔGWT), which accounts for contributions from hydrophobic, polar, van der Waals, hydrogen bonding, and electrostatic interactions.

### Data analysis and statistics

Data analysis was performed using GraphPad Prism 9 (GraphPad Software, San Diego, CA, USA, www.graphpad.com), and results are expressed as mean values ± standard deviation (SD) unless otherwise specified. To study the replication kinetics of viral variants, ΔCt was calculated by subtracting the cycle threshold (Ct) values of non-infected cells (used as control) from those of infected cells. The AUC was then calculated, and nonlinear logistic growth analysis was applied to model the kinetic replication curve.

For the immune activation analysis, ΔCt was calculated by subtracting the Ct of the housekeeping gene from the Ct of the target gene for each sample. Statistical analysis for both replication kinetics and immune activation was conducted using two-way analysis of variance (ANOVA) followed by Tukey’s multiple comparisons test.

To assess the inhibition of pseudoviral entry, transduction data were normalized to uninfected cells (set to 100%) and untreated infected cells (set to 0%). The CPE in Vero E6/TMPRSS2 and MDCK cells was normalized to virus infection controls (set to 0% inhibition) and uninfected cells (set to 100% inhibition). These data were analyzed using two-way ANOVA and Tukey’s multiple comparisons test.

To evaluate the relationships between different variables, two separate Spearman’s rank correlation analyses were performed. In the first analysis, Spearman’s correlation was used to explore the relationships between viral entry mechanisms, replication kinetics, and immune activation. The correlation coefficients (*r*) were calculated for each pairwise comparison, with values ranging from −1 (perfect negative correlation) to +1 (perfect positive correlation), and a coefficient of 0 indicating no correlation. In the second analysis, Spearman’s correlation was applied to assess the relationship between viral persistence data and the stability of viral spike proteins, where the stability was quantified based on ΔΔG (DDG) values obtained through computational analysis. This analysis allowed us to examine whether viral persistence could be linked to the stability of the spike variants, providing insight into potential structural determinants of viral persistence. Statistical significance was considered for both analyses at *P* < 0.05.

For the evaluation of the functional mutation G446D in pseudovirus assays, the Mann-Whitney U-test was applied to compare the persistence data between infected and uninfected groups.

## RESULTS

### Insights into the replication kinetics of 11 SARS-CoV-2 variants

**Fig 1:**
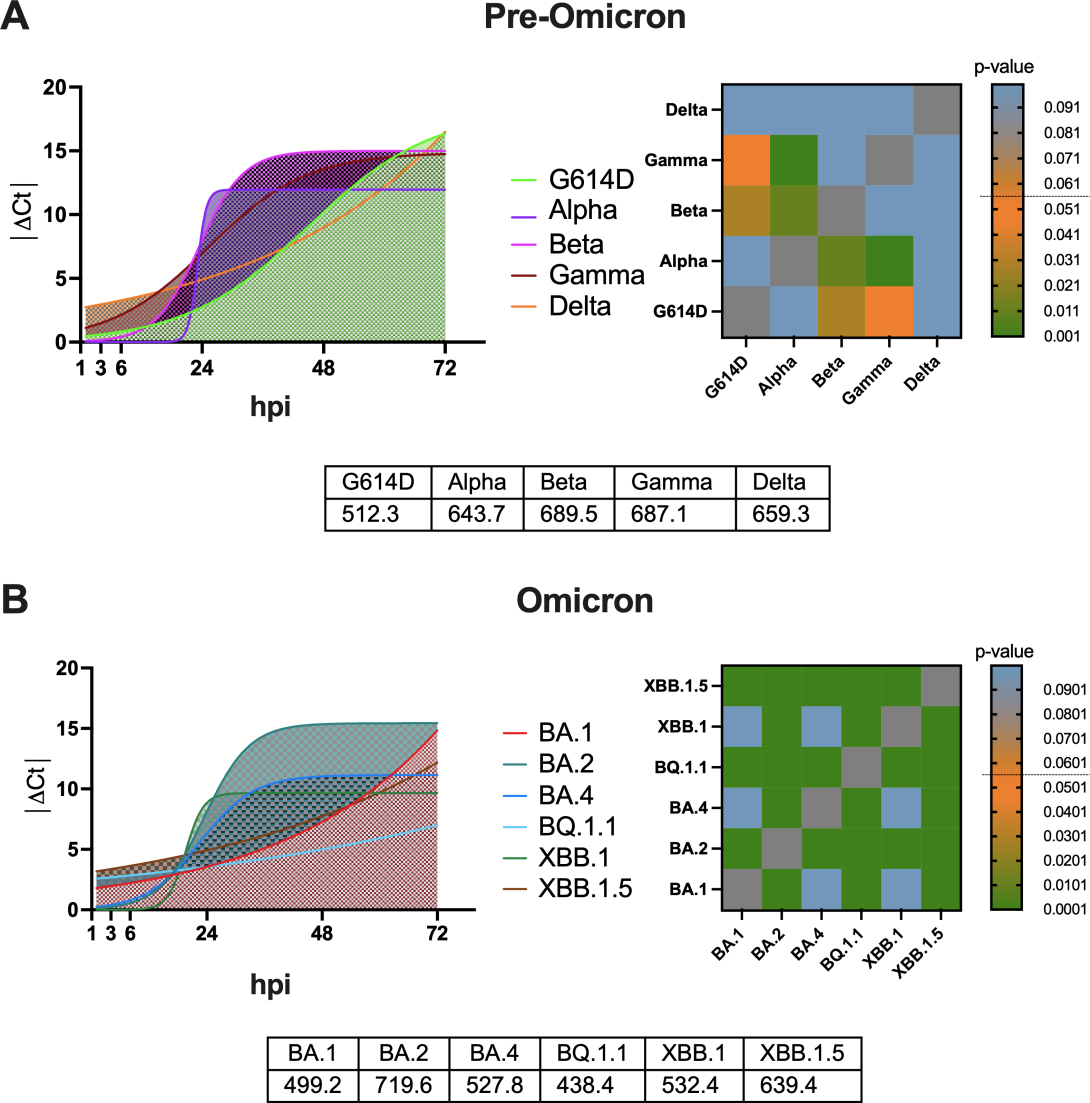
Replication kinetics of 11 SARS-CoV-2 VOCs in Calu-3 cells. Viral genome copy numbers in the supernatant were measured at six time points post-infection (0.001 MOI). (**A**) Pre-Omicron VOCs: G614D, Alpha, Beta, Gamma, and Delta replication kinetics. Statistically significant differences were observed between Beta/Gamma and G614D/Alpha (**P < 0.01). (**B**) Omicron family variants: BA.1, BA.2, BA.4, BQ.1.1, XBB.1, and XBB.1.5 were assessed for viral replication. Significant differences were observed between BA.2, BQ.1.1, and XBB.1.5 (***P < 0.001), with no significant differences among BA.1, BA.4, and XBB.1 (*P* > 0.1). The viral RNA levels were quantified by real-time PCR and expressed as ΔCt values, with mean ± SD for each variant. Statistically significant differences in replication kinetics were determined, with **P < 0.01, ***P < 0.001, and ****P < 0.0001. Ct: threshold cycle.

We conducted an analysis of the replication kinetics of 11 SARS-CoV-2 variants, including pre-Omicron variants (G614D, Alpha, Beta, Gamma, and Delta) and Omicron sublineages (BA.1, BA.2, BA.4, BQ.1.1, XBB.1, and XBB.1.5). These variants were classified as VOCs by the WHO over time but are currently being de-escalated due to their decline in circulation as reported by the European Centre for Disease Prevention and Control (https://www.ecdc.europa.eu/en/covid-19/variants-concern). To evaluate the viral replication dynamics, human lung epithelial carcinoma cells (Calu-3) were used as a surrogate model for pulmonary infection. The results demonstrate that replication kinetics varied significantly among the SARS-CoV-2 variants, with distinct viral RNA levels detected at multiple time points. These differences were reflected in the AUC values, indicating variant-specific replication profiles. Notably, viral RNA copy numbers in the supernatant increased rapidly during the first 24 hpi and subsequently plateaued around 72 hpi. Focusing on the pre-Omicron family variants (Fig. 1A), Beta and Gamma variants demonstrated the fastest replication kinetics, with very similar AUC values (689.5 and 687.1, respectively), followed by Delta (AUC value 659.3) and Alpha (AUC value 643.7), and finally, G614D exhibited the slowest replication rate, with a small AUC value (512.3). Statistically significant differences in replication kinetics were observed comparing Beta and Gamma to G614D and Alpha (*P* < 0.05). No statistically significant differences were found comparing the Delta with the other variants.

For the Omicron family variants (Fig. 1B), BA.2 exhibited the fastest replication kinetics (AUC value 719.6), followed by XBB.1.5 (AUC value 639.4). The BA.1, BA.4, and XBB.1 variants showed similar AUC values (499.2, 527.8, and 532.4, respectively), all higher than BQ.1.1, which displayed the lowest replication rates (AUC value 438.4). Statistical analysis revealed significant differences in replication rates for BA.2, BQ.1.1, and XBB.1.5, compared to all other VOCs, while no significant differences were observed when comparing the replication rates of BA.1, BA.4, and XBB.1.

### Different replication kinetics could be influenced by a different entry mechanism

**Fig 2:**
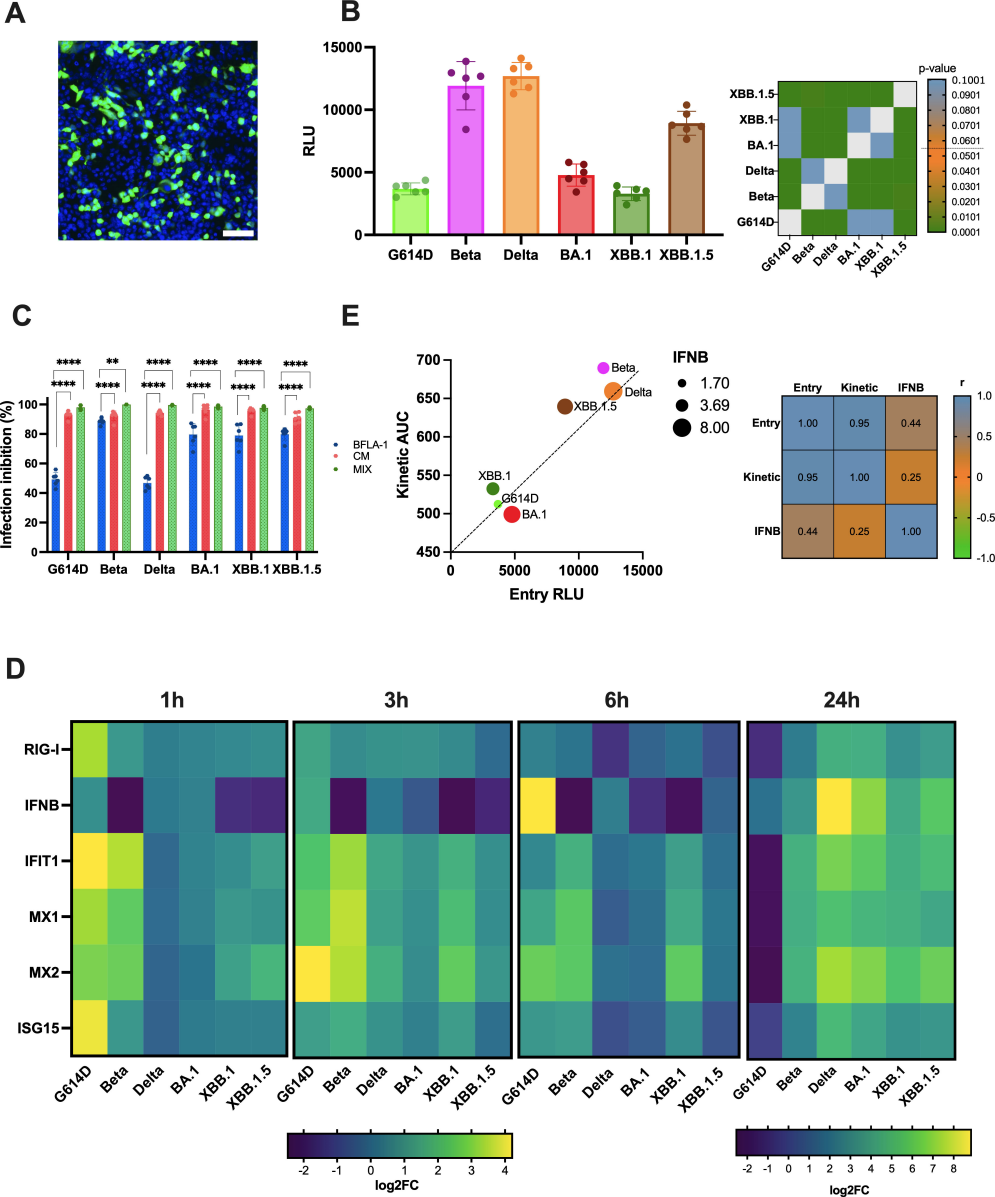
Impact of entry mechanisms and immune response on the replication kinetics of 11 SARS-CoV-2 VOCs. To investigate how entry mechanisms influence the replication kinetics of SARS-CoV-2 VOCs, we used lentiviral-based pseudovirus system expressing full-length S protein and ZsGreen or luciferase for infection monitoring. (**A**) Assessment of pseudovirus production was evaluated by ZsGreen signal in HEK 293T-ACE2/TMPRSS2-positive cells using 20× magnification. Nuclei were counterstained with Hoechst 33342 (blue). Scale bar, 100 µm. (**B**) Entry experiments were performed using the pseudovirus carrying different S proteins to infect Calu-3 cells at equal doses, and luciferase activity (RLU) was detected after incubation at 37°C for 72 h. Data from six replicates are shown as the mean ± SD, **** *P* < 0.0001. (**C**) Mechanisms used by the virus to infect Calu-3 through endocytosis and direct fusion with plasma membrane were selectively hampered by chemical compounds. Targeting only one of the two entry mechanisms resulted in different degrees of infection inhibition. The infection inhibition percentages were reported as mean values ± SD, ** *P* < 0.01, ****, *P* < 0.0001. (**D**) Heatmap showing the activation of the interferon signaling pathway following infection with 0.1 MOI. The activation (value > 1) or downregulation (value < 1) of IFN-related genes was calculated by subtracting the Ct values of specific genes from the Ct values of the housekeeping gene (GAPDH), yielding the ΔCt. Gene expression is presented as log2 fold change relative to uninfected cells (ΔΔCt). Monitoring the immune response at different time points provided insights into how specific VOCs may mediate distinct activation or suppression paths. (**E**) Correlation analysis was performed on 11 VOCs, considering entry values (*x*-axis), replication kinetics (*y*-axis), and immune response (size). Entry values are indicated by the RLU signal, replication kinetics are represented by the AUC value, and immune escape is reflected by the upregulation of IFN-β levels at 24 hpi. Pearson correlation analysis is shown in the heatmap, highlighting a strong correlation between entry and replication mechanisms (0.95).

We set up a lentiviral-based pseudoviruses protocol for three pre-Omicron family (G614D, Beta, Delta) and three Omicron family (BA.1, XBB.1, and XBB.1.5), as described in the supplementary section. Initial validation confirmed efficient infection of 293T-ACE2/TMPRSS2 cells by the G614D pseudovirus (Fig. 2A) and successful pseudovirus production in VeroE6/TMPRSS2 cells (Fig. S1).

To confirm consistency, we performed protein quantification by bicinchoninic acid (BCA) assay confirming equal total protein levels across pseudovirus preparations (Fig. S2). Subsequent enzyme-linked immunosorbent assay (ELISA) demonstrated comparable S protein expression among variants (Fig. S3).

Using equivalent amounts of pseudovirus normalized by ELISA optical density, entry assays in Calu-3 cells revealed variant-specific differences in viral entry efficiency.

The obtained results (Fig. 2B) illustrate that the VOCs exhibit distinct entry mechanisms. Specifically, the Beta and Delta variants displayed the fastest entry kinetics (*P* < 0.0001), followed by XBB.1.5 (*P* < 0.0001). In contrast, the G614D, BA.1, and XBB.1 variants showed comparable RLU signals, suggesting similar entry profiles. Statistical analysis further confirmed that no significant differences were observed between Beta and Delta, nor between G614D, BA.1, and XBB.1. However, the entry kinetics of XBB.1.5 were significantly different from all other variants (*P* < 0.0001).

To deeply discover the entry process, we examined the involvement of the early and the late pathways for all the lentiviral-based pseudoviruses, evaluating the inhibitory effect of BFLA-1 and CAM, alone or combined. As shown in the graph (Fig. 2C), a significant inhibitory effect was observed with CAM treatment (93.6 ± 2.4% of infection inhibition) across all pseudoviruses, while inhibition of endosomal acidification by BFLA-1 only partially hindered pseudovirus entry (70.5 ± 17.4% of infection inhibition). This result was particularly evident in G614D and Delta pseudoviruses (48.5 ± 2.12) and suggested that all the SARS-CoV-2 VOCs examined mainly relied on early pathway if TMPRSS2 was expressed on the host cell membranes. The combination of both inhibitors led to 99.9 ± 0.1% of inhibition, suggesting a complete hampering of the viral entry step.

### Immuno-escape: how the antiviral response impacts the replication kinetics

We then focused on the role of the innate immunity of the Calu-3 cell lines, exploring how this may influence both these virological aspects. Therefore, we evaluated the first host defense against virus infection, the type I interferon (IFN) response released by cells through a signaling cascade started with the activation of RIG-I or RIG-I-like receptors (RLRs) upon interaction with viral products.

The gene expression results showed (Fig. 2D) how the mRNA levels of IFN-β and the ISGs significantly increased upon SARS-CoV-2 infection in Calu-3 compared to uninfected control. Specifically, considering the immune response after 1 hpi, we observed that G614D was rapidly recognized, triggering an immune response as demonstrated by the upregulation of mRNA levels of both pattern recognition receptors (PRR) and ISGs with statistically significant differences (*P* < 0.001) when compared to the other variants (Fig. S4 and S5). The IFN-β response was similar between G614D, Delta, and BA.1 and downregulated in Beta (*P* < 0.0001), XBB.1 (*P* < 0.01), and XBB.1.5 (*P* < 0.001) compared to the other variants (Fig. S4). Notably, at 3 hpi with G614D, the levels of RIG-I and ISG15 decrease and return to baseline, with no significant difference compared to the others. In contrast, IFN-β expression was significantly elevated at 3 hpi in both G614D and Delta variants compared to other VOCs. Specifically, G614D showed statistically significant differences compared to Beta, XBB.1 (*P* < 0.0001), BA.1 (*P* < 0.01), and XBB.1.5 (*P* < 0.001), while Delta exhibited significant differences when compared to Beta (*P* < 0.01) and XBB.1 (*P* < 0.001). G614D reached its peak expression at 6 hpi (*P* < 0.0001). Moreover, at 6 hpi, an upregulation of downstream genes involved in the interferon response was observed in Beta and XBB.1 compared to the other variants (*P* < 0.0001).

At the 24 h time point, G614D exhibited a downregulation of IFN-β and ISGs (*P* < 0.0001), which may correlate with the resolution of the infection.

In contrast, all other variants displayed an increased expression of IFN-β (*P* < 0.0001). Notably, Beta did not exhibit further upregulation of RIG-I, IFIT1, MX1, MX2, and ISG15 compared to earlier time points, whereas the levels of these genes continued to rise in all other VOCs (*P* < 0.0001).

Considering the three different virological aspects, we conducted a multivariable analysis to identify potential correlations (Fig. 2E). The obtained results allow us to conclude that the viral entry mechanisms and replication kinetics are strongly correlated (*r* = 0.95): variants with faster entry also exhibit higher replication kinetics. However, when considering the abundance of the IFN response at 24 hpi in relation to viral replication and entry mechanisms, no significant correlation was observed (*r* < 0.5).

### Study the persistence mechanisms of SARS-CoV-2 variants on different materials

We aimed to study transmission through contaminated objects: fomites. We set up an experimental protocol to investigate the persistence of 11 SARS-CoV-2 variants on three selected materials: plastic, aluminum, and copper.

**Fig 3:**
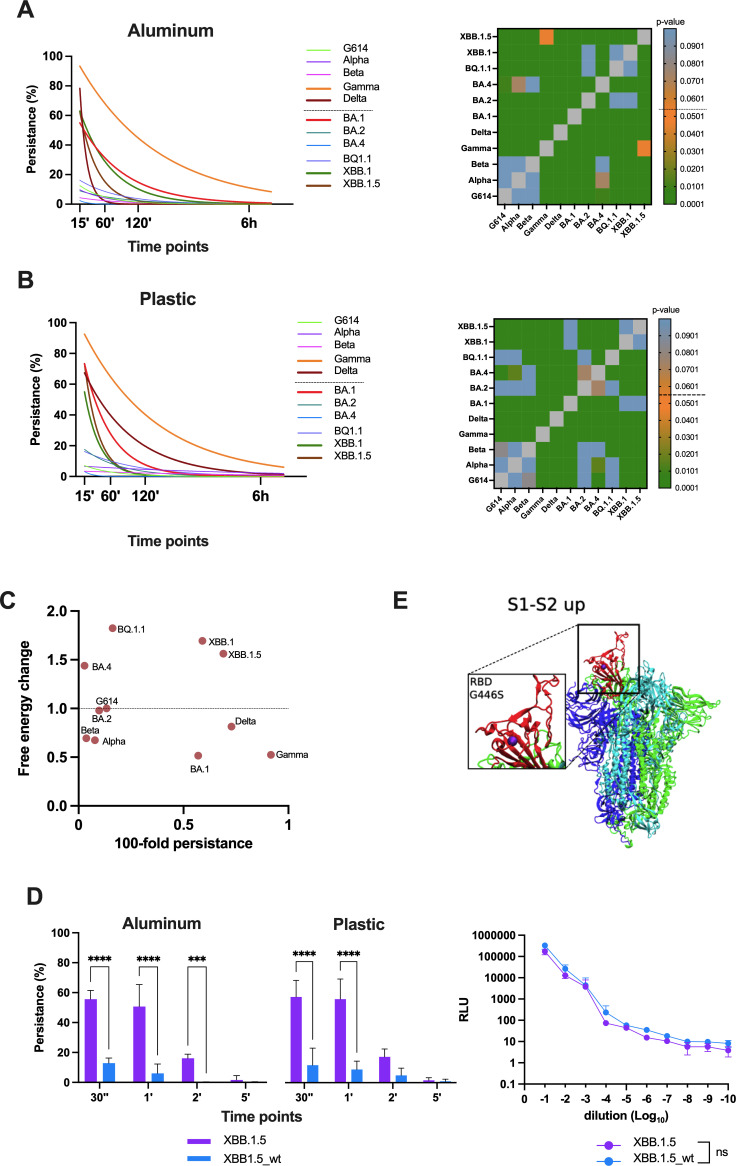
Persistence mechanism as an alternative transmission route: the role of the G446S mutation. (**A, B**) Fomite transmission was studied on plastic and aluminum surfaces at different time points. The persistence percentage was calculated based on the detected viral load (TCID_50_ per milliliter) at each time point, relative to the titer of viral stock (TCID_50_ per milliliter). The decrease in viral load was estimated by fitting a logistic growth curve. Data are shown as the mean ± SD, ****P < 0.0001. (**C**) The graph shows persistence value (*x*-axes) expressed as 100-fold and the DDG obtained from MAESTRO analysis (*y*-axis) and expressed as 2^DDG^, considering the value from 0 to 1 spike conformation with higher stability, while values from 1 to 2 spike conformations with lowest stability. (**D**) Pseudovirus particles carrying XBB.1.5 or XBB.1.5_wt S proteins were used to infect Calu-3 cells, and luciferase activity (RLU) was measured after 72 h of incubation at 37°C. Data from six replicates are presented as mean ± SD and are not statistically different (*P* = ns). An equal amount of pseudoviruses was used in persistence experiments, where the recovered pseudoparticles were used to infect Calu-3 cells, and RLU signals were measured after 72 h of incubation at 37°C. The data are presented as mean and ± SD, and *t*-test analysis was performed, **P < 0.01, ****P < 0.0001. (**E**) Representative structure of the G614D spike protein in the 1-up state to map the location of the G446S mutation within the RBD domain and assess its potential role in increased persistence.

The obtained results allowed us to draw two main observations: none of the tested variants were able to persist for more than 15 min on copper (Fig. S6), a material known for its antimicrobial and antiviral properties. For plastic and aluminum, which are non-porous and non-charged materials, we observed that some variants were able to persist (Fig. 3A and B). The persistence effect is not material but variants-dependent: the same variants that persist remain stable on both materials. At the first time point of 15 min, Gamma was the most persistent variant, with a detected percentage of viral load of 91.6 ± 2.8% on aluminum and plastic, followed by Delta with a viral load persistence of 78.3 ± 5.7% on aluminum and 67.3 ± 2.5% on plastic. Among the Omicron variants, BA.1, XBB.1, and XBB.1.5 exhibited similar persistence values; the detected viral load of BA.1 was 55 ± 1% on aluminum and 73.3 ± 2.8% on plastic, while for the XBB variants, it was 62.6 ± 8.08% on aluminum and 64.2 ± 10.7% on plastic. After 120 min, Gamma showed a persistence rate of 81.6 ± 6.3% on aluminum and 43.3 ± 2.8% on plastic, while Delta showed 20 ± 5% persistence on plastic. The BA.1 and XBB.1 variants had 15 ± 2.6% persistence on plastic, while only BA.1 persisted on aluminum with 7.6 ± 4%. XBB.1.5 did not persist further. Lastly, after 6 h, no variants remained detectable on either material. We performed a two-way ANOVA to analyze the variance in persistence mechanisms at 15 min for all VOCs across the two materials. The persistence rates of the variants were significantly different, with a *P*-value <0.01 compared to other VOCs as shown in the heatmap.

### Implication of different mutations in the persistence mechanism

To investigate the impact of mutations on the persistence mechanisms of SARS-CoV-2 variants, we performed a variant calling analysis on the S, E, and M proteins. The mutations identified, summarized in Table 3, are based on a prior structure-based analysis: bold indicates stabilizing mutations, bold-italics denotes destabilizing mutations, and bold-underlined represents mutations not classified for stability effects (12).

**TABLE 3:** Variant calling analysis on the entire genome of the 11 VOCs^*a*^

	S1			S2	E	M
	NTD	RBD				
Alpha	del 69-70; del 144	***N501Y***; A570D; P681H		T716I; S982A; D1118H		
Beta	**L18F**; D80A; ***T95I***; D215G; del 241-242	***K417N***; E484K; ***N501Y***		**A701V**		
**Gamma**	**L18F;** T20N; ***P26S***; **D138Y**; R190S	K417T; E484K; ***N501Y***	**H655Y**	**T1027I**; V1176F	**P71L**	
**Delta**	T19R; E156G; del 157-158	***L452R***; T478K; P681R		D950N; D1084H		**I82T**; S94G
**BA.1**	**A67V**; del 69-70; ***T95I****; **G142D***; del 143-145; del 211; L212I; ins EPE	G339D; S371L; S373P; S375F; ***K417N***; N440K; **G446S**; **S477N**; T478K; E484A; Q493R; G496S; Q498R; ***N501Y***; Y505H; T547K	**H655Y;** N679K; **P681H**	**A701V;** N764K; **D796Y**; N856K; Q954H; N969K; L981F	**T9I**	D3G; Q19E; A63T
BA.2	T19I; del 24-26; A27S; ***G142D***; V213G	G339D; S371F; S373P; S375F; T376A; D405N; R408S; ***K417N***; N440K; **S477N**; T478K; E484A; Q493R; ***N501Y***; Y505H	**H655Y;** N679K; **P681H**	N764K; **D796Y**; Q954H; N969K	**T9I**	Q19E; A63T
BA.4	T19I; del 24-26; A27S; del 69-70; ***G142D***; V213G	S371F; S373P; S375F; T376A; D405N; R408S; ***K417N***; N440K; T478K; E484A; F486V; Q498R; ***N5s01Y***; Y505H	**H655Y;** N679K; **P681H**	N764K; **D796Y**; Q954H; N969K	**T9I**	D3N; Q19E; A63T
BQ.1.1	T19I; del 24-26; A27S; del 69-70; ***G142D***; V213G	G339D; R346T; S371F; S373P; S375F; T376A; D405N; R408S; ***K417N*;** N440K; K444T; ***L452R***; E484A; F486V; Q498R; ***N501Y***; Y505H	**H655Y;** N679K; **P681H**	N764K; **D796Y**; Q954H; N969K	**T9I**	D3N; Q19E; A63T
**XBB.1**	T19I; del 24-26; A27S; V83A; ***G142D***; del 144; V213G; G252V	G339H; R346T; S371F; S373P; S375F; T376A; D405N; R408S; ***K417N***; N440K; V445P; **G446S**; T478K; E484A; F486V; F490S; Q498R; ***N501Y***; Y505H	**H655Y;** N679K; **P681H**	N764K; **D796Y**; Q954H; N969K; S982A	**T9I**	Q19E; A63T
**XBB.1.5**	T19I; del 24-26; A27S; V83A; ***G142D***; del 144; V213G; G252V	G339H; R346T; S371F; S373P; S375F; T376A; D405N; R408S; ***K417N***; N440K; V445P; **G446S**; T478K; E484A; F486P; F490S; Q498R; ***N501Y***; Y505H	**H655Y;** N679K; **P681H**	N764K; **D796Y**; Q954H; N969K; S982A	**T9I**	Q19E; A63T

^*a*^ Boldface type indicates stabilizing mutations, boldface and italic type denotes destabilizing mutations, and boldface type with underlining represents mutations not classified for stability effects.

We then focused on the mutations located in the exposed domains of the S protein (S1 subunit, receptor-binding domain, and S2 subunit) to assess their impact on the overall stability of the protein. The obtained results highlighted a significant shift in the stability of the S protein during the SARS-CoV-2 evolution. Among the pre-Omicron variants, the G614D S protein exhibited the lowest stability (0.97 kcal/mol, *r* = 0.817), whereas the Gamma variant displayed the highest stability (0.52 kcal/mol, *r* = 0.885), consistent with the results of the persistence experiment where the viral load of the Gamma remained more stable over time compared to the other variants (Fig. 3C). The stability of the Alpha, Beta, and Delta variants was similar (0.67 kcal/mol, 0.69 kcal/mol, and 0.81 kcal/mol, respectively) with *r* = 0.8; however, the Delta variant exhibited a longer persistence compared to Alpha and Beta. In the Omicron family, it is noteworthy that the BA.1 variant, the most stable among the BA lineage (0.51 kcal/mol, *r* = 0.79), showed the most stable conformation. In contrast, subsequent Omicron variants exhibited either positive stability values or values close to 1, suggesting a more unstable S conformation (BA.2: 0.93 kcal/mol, *r* = 0.86; BA.4: 1.43 kcal/mol, *r* = 0.80; BQ.1.1: 1.82 kcal/mol, *r* = 0.75; XBB.1: 2.69 kcal/mol, *r* = 0.73; XBB.1.5: 2.56 kcal/mol, *r* = 0.74). This observation further supports the persistence of BA.1, indicating a more stable structural conformation that may contribute to its persistence over time. Conversely, the XBB variants, which show greater persistence than other BA variants, display increased instability, likely due to the mutations present in their S proteins. The obtained results suggest that the mutations influencing the stability of the S protein may also contribute to mechanisms of persistence.

Focusing on the variant calling table (Table 3), the persistence variants (Gamma, Delta, BA.1, XBB.1, and XBB.1.5) exhibited stabilized and destabilized mutations. Gamma carried two destabilizing and five stabilizing mutations, including *D138Y* and *T1027I* in the S protein domains, which were not conserved. Delta had one destabilizing mutation and a single stabilizing mutation, *I82T* in the E domain, which was not present in other variants. BA.1 showed two destabilizing mutations and seven stabilizing mutations (*A67V, S477N, H655Y, P681H, A701V,* and *D796Y* on the S and *T9I* on the E), all of which were also found in non-persistent variants. Similarly, XBB.1 and XBB.1.5 each had three destabilizing mutations and four stabilizing mutations (*H655Y, P681H,* and *D796Y* on the S and *T9I* on the E), which were already present in non-persistent variants. The only mutation conserved across all persistent Omicron variants was G446S in the RBD domain. The role of this mutation in protein stability had not been characterized in previous studies, although the G446V mutation in the same region was identified as stabilizing (12). Moreover, considering the virological significance of the G446S mutation, particularly its role in immune escape and neutralization sensitivity, we further investigated its function using a lentiviral pseudovirus approach (13).

Starting with the XBB.1.5 mutant S protein, we reverted the mutation to the wild-type and we evaluated the titer of both wild-type and mutated pseudoviruses shown in the corresponding graph (Fig. 3D). No statistically significant difference was observed between the two pseudoviruses. We then performed the persistence experiment, and the obtained data confirmed that the pseudovirus carrying the wild-type S mutation exhibits significantly lower persistence compared to the G446S pseudovirus.

At the first time point (30 s), the mutated pseudovirus exhibited a persistence percentage of 56.6 ± 12% on plastic and 55.6 ± 5% on aluminum, while the wild type showed a persistence percentage of 10.7 ± 3.7% on plastic and 12.8 ± 3% on aluminum. After 1 min, no significant difference in persistence percentage was observed; at 2 min, the G446S pseudovirus exhibited a persistence percentage of 16.7 ± 3.8%, while the wild-type dropped to less than 5% on plastic and was completely absent on aluminum. At both 5 and 30 min, no detectable pseudovirus load was observed on either surface. These findings suggest that the G446S mutation may enhance the stability of the spike protein, potentially playing a key role in the persistence mechanism of the pseudovirus.

To further validate the role in the persistence mechanism, we performed molecular modeling, and the obtained structure revealed that the mutation occurs in an exposed region of the RBD, which may facilitate interactions with surfaces, potentially enhancing persistence (Fig. 3E).

### Evaluating the efficacy of “green” disinfectants against persisting viral variants

As a final aspect, we assessed the disinfectant efficacy of persistent variants using naturally derived disinfectants such as TTO and QRE, as well as biomolecules such as DAP.

**Fig 4:**
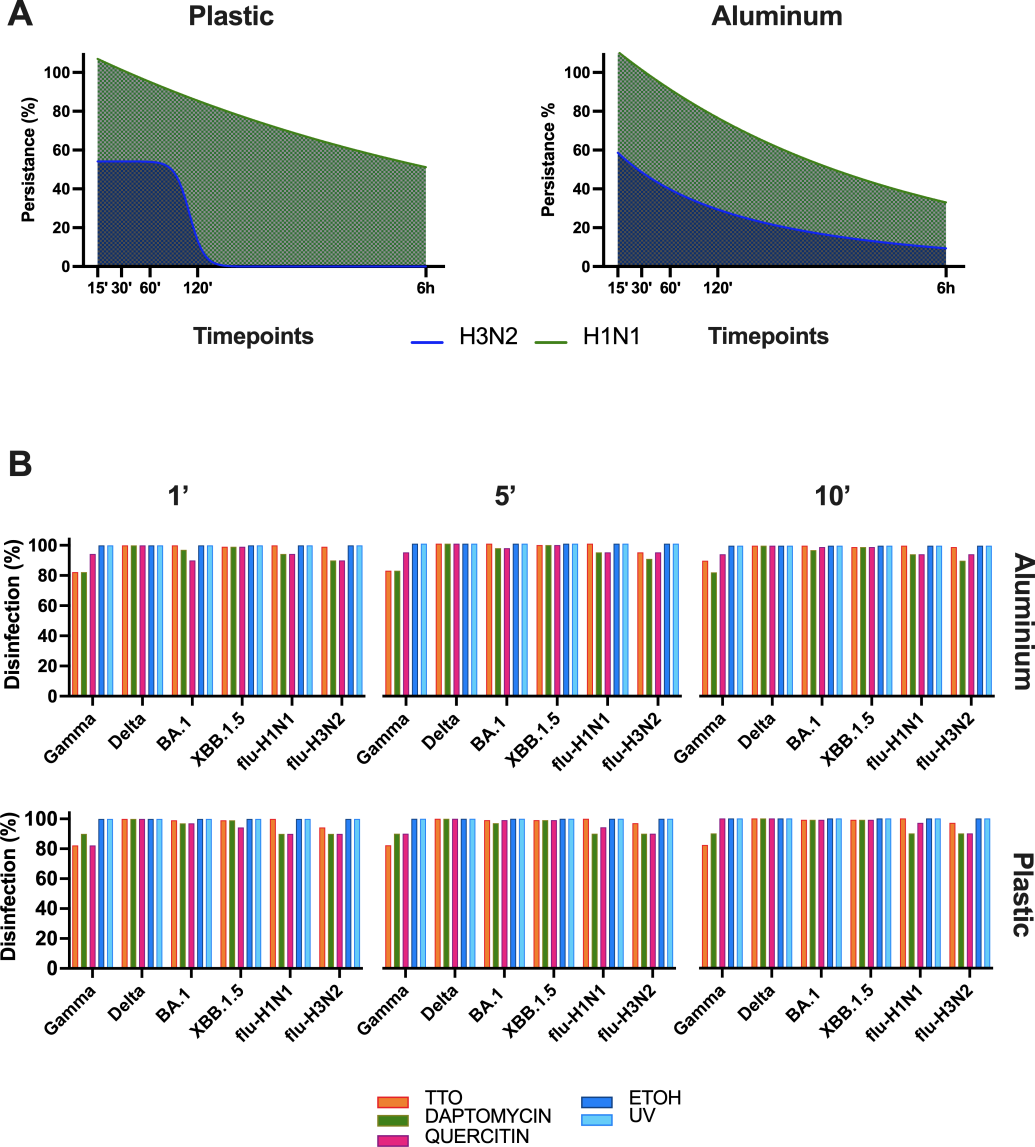
Assessment of disinfectant efficacy against persistent SARS-CoV-2 variants and influenza strains. (**A**) Persistence assays were calculated by infecting MDCK cells with recovered viral particles, and viral load was expressed as the fold change in TCID_50_ per milliliter relative to the starting stock titer. The decrease in viral load was estimated by fitting a logistic growth curve. Data are shown as the mean ± SD. (**B**) The efficacy of disinfectants, including TTO, QRE, DAP, and two positive controls (alcohol and UV-C), was tested on plastic and aluminum surfaces at 1, 5, and 10 min time points. All disinfectants reduced viral load by up to 80% after 1 min. TTO, DAP, and QRE achieved >95% reduction in viral load for SARS-CoV-2 variants, with TTO being the most effective on aluminum (1.88 ± 0.36%) and plastic (8.4 ± 2.08%) at 1 min. Alcohol and UV-C achieve complete disinfection within 1 min.

Based on the persistence assay results, two influenza A strains (H1N1 and H3N2) had longer persistence compared to SARS-CoV-2 variants and were thus selected as positive controls. The H1N1 strain exhibited 100% persistence up to 120 min and maintained over 10% persistence at 6 h on all surfaces except copper. In contrast, the H3N2 strain showed approximately 50% persistence at 60 min, which decreased to below 10% at 120 min and 6 h across all tested surfaces. These persistence profiles justified their use as positive controls in the evaluation of disinfectant efficacy (Fig. 4A).

For the assessment of disinfectant activity, we repeated the persistence experiment using the Gamma, Delta, BA.1, and XBB.1 variants, with H1N1 and H3N2 strains evaluating the antiviral efficacy of the compounds within a 15 min persistence window, with time points at 1, 5, and 10 min, on both plastic and aluminum surfaces (Fig. 4B).

We can conclude that all disinfectants demonstrate a broad spectrum of antimicrobial activity, effectively achieving up to 80% reduction in viral load after just 1 min of exposure. Excluding the Gamma variant, all other SARS-CoV-2 variants and influenza strains were disinfected by more than 95% using TTO. Specifically, on aluminum, the percentage of residual viral load detected at 1 to 10 min was 1.88 ± 0.36%, while on plastic, the residual percentage was 8.4 ± 2.08% at 1 min, decreasing to 5.9 ± 1.19% at both 5 and 10 min. The disinfection efficacy of DAP and QRE was higher for SARS-CoV-2 variants compared to influenza strains. For SARS-CoV-2 VOCs, the residual percentage of viral load after 10 min of treatment with DAP was 1.38 ± 1.29% on aluminum and 0.66 ± 0.47% on plastic. In contrast, for influenza strains, the percentage of residual viral load was 7.81 ± 2.19% on aluminum and 10.00% on plastic.

After treatment with QER, the percentage of residual viral load for SARS-CoV-2 VOCs was 0.92 ± 0.72% on both aluminum and plastic surfaces. In comparison, influenza strains showed a percentage of residual viral load of 5.62% on aluminum and 6.58 ± 3.42% on plastic. Gamma, unlike the other variants, was more effectively disinfected by QER, with only 17% of the viral load detected on aluminum and complete disinfection on plastic after 10 min. In contrast, TTO and DAP exhibited similar residual viral loads after 10 min, with 10% detected on metal and 17% on plastic. Notably, both positive controls (alcohol and UV-C) were able to achieve complete disinfection (100%) of all viruses after just 1 min of treatment.

## DISCUSSION

The evolution of SARS-CoV-2 has produced variants with distinct replication, immune evasion, and transmission traits (14). This study analyzes 11 variants, including pre-Omicron and Omicron sublineages, revealing key differences in their biological behavior and public health implications.

We confirmed that pre-Omicron variants (Beta, Gamma, Delta) showed faster replication, suggesting mutations enhanced viral fitness (15, 16). In contrast, Omicron variants (BA.1, XBB.1, XBB.1.5) replicated more slowly, likely due to S protein mutations favoring mucosal transmission over lung infection (17, 18). These findings suggest specific mutations optimize viral entry and replication, influencing disease severity and transmission dynamics. To investigate the impact of mutations on the entry process, we employed a lentiviral pseudovirus system expressing variant-specific S proteins. The results demonstrated a clear correlation between replication kinetics and entry efficiency, with faster-replicating variants showing more efficient entry. To further dissect entry dynamics, we treated Calu-3 cells with two inhibitors targeting distinct viral entry pathways: BFLA-1 and CAM. Notably, BFLA-1, a vacuolar H^+^-ATPase inhibitor, prevented the cleavage of the S protein and exposure of the fusion peptide (FP) domain by disrupting the acidic environment required for cathepsin L activation, while CAM targeted the early pathway by inhibiting TMPRSS2 activity and preventing direct fusion between the host membrane and the viral envelope (19). The data revealed that inhibiting TMPRSS2 activity had a more pronounced effect on viral entry across all variants compared to targeting endosomal acidification (20). Delta and Beta variants, with faster replication and entry, were over 90% inhibited with CAM but less than 50% with BFLA-1, indicating a preference for direct fusion and higher transmissibility. Omicron variants, over 80% inhibited with BFLA-1, rely on the late entry pathway, likely due to spike conformational changes. The G614D variant, despite slower entry, showed only 50% BFLA-1 inhibition, suggesting reduced spike cleavage affects TMPRSS2 interaction and viral entry dynamics.

We also evaluated the immune response by assessing viral escape through the first line of defense of the host: the IFN-I response. IFN plays a critical role in the early detection of viral infections, activating PRRs that detect viral RNA and initiate antiviral immune responses (21). This triggers a cascade of antiviral mechanisms, including innate immune cell activation and the creation of an environment that restricts viral replication (22). Upon detection of viral RNA by PRRs like RIG-I, signaling pathways activate transcription factors to induce IFN-β expression, which binds to the IFN-α/β receptor and activates the JAK-STAT pathway, driving the expression of ISGs like IFIT1, MX1, MX2, and ISG15 to inhibit viral replication and enhance immune responses (23).

Our results confirmed that SARS-CoV-2 variants elicit distinct IFN signatures in Calu-3 cells. G614D induced strong early immune activation, consistent with a reduced replication rate (24). In contrast, Beta and XBB.1 showed delayed or weak IFN-β responses, supporting immune evasion mechanisms already described (16, 25). Omicron variants activated IFN-I at later time points, aiding persistence. Sustained IFN-β activation was also seen in Delta, indicating better immune recognition and possible control of viral replication (26). These findings underline how differential IFN responses among variants influence replication, immune evasion, and overall infectivity (23).

To evaluate the transmissibility of SARS-CoV-2 variants, we examined their stability on surfaces, which is crucial for transmission, especially indoors and in healthcare settings where fomites can contribute to indirect infection. While respiratory droplets are the primary transmission route, contact with contaminated surfaces remains a risk. We tested the persistence of SARS-CoV-2 on plastic, aluminum, and copper, materials with distinct properties that affect viral stability. Aluminum and copper are non-ferrous metals, while plastic is a durable polymer, influencing virus retention. These material differences—such as conductivity and surface charge—affect virus persistence, highlighting the role of surface composition in transmission risk. Our results confirmed that copper inactivated the virus within 15 min, while plastic and aluminum supported longer viral persistence.

Copper exhibited strong antiviral properties, rapidly inactivating the virus within 15 min, whereas plastic and aluminum supported longer viral persistence, particularly for variants like Gamma and Delta, which showed greater stability over time. This finding aligns with previous studies showing SARS-CoV-2 can persist on non-porous surfaces for up to 72 h (27, 28). Our analysis suggested that viral persistence is more dependent on viral characteristics than surface material, with Gamma, Delta, BA.1, XBB.1, and XBB.1.5 exhibiting the highest stability.

To deeply investigate the persistence mechanism, we analyzed the stability of the spike protein using the ∆∆G metric, which measures stability changes due to mutations. The analysis confirms an evolutionary stability shift in the SARS-CoV-2 variants: pre-Omicron like Gamma and Delta had stability values near zero, indicating stable spike proteins (12). Omicron BA.1 had similar stability, but the XBB variants, which persisted longer on surfaces, showed higher ∆∆G values, suggesting instability. We further expanded our analysis to include variant calling and identified a conserved mutation, G446S, in persistent Omicron variants. Located in the RBD, this mutation may enhance surface interactions, contributing to the environmental persistence of the virus. To investigate the role of the G446S mutation in persistence, we reverted it to the wild-type form and tested persistence using a variant-specific S pseudovirus model. The results showed that the wild-type pseudovirus persisted less on surfaces than the one with the G446S mutation. This suggests that G446S may enhance the ability of the virus to remain infective on surfaces for longer periods.

We also tested the disinfectant activity of natural compounds like TTO, QRE, and DAP, which demonstrated significant antiviral effects. TTO inhibited the spike protein-ACE2 interaction, while QRE, a flavonoid, reduced viral infection by binding key SARS-CoV-2 proteins (29, 30). DAP, a cyclic lipopeptide, showed antiviral properties by disrupting viral envelope formation (31). These compounds reduced viral load by up to 90% after 10 min of exposure, outpacing common disinfectants like UV-C light and alcohol-based solutions, which inactivated the virus within 1 min (23). While UV-C and alcohol are effective, they pose health and environmental risks, making natural compounds like TTO, QRE, and DAP viable, non-toxic alternatives for disinfection.

In conclusion, this study aims to provide a comprehensive analysis of the replication kinetics and immune evasion properties of 11 SARS-CoV-2 VOCs, with a particular focus on their persistence on surfaces and response to various disinfectants compared to other respiratory viruses such as influenza A. Furthermore, whole-genome sequencing and *in silico* modeling enabled the identification of mutations involved in virological mechanisms, validating the *in vitro* results obtained from the persistence experiment. The findings highlight the importance of continued surveillance and timely disinfection to control transmission. Natural disinfectants like TTO and QRE, alongside conventional methods, offer effective, complementary strategies for infection control considering their broad activity on different respiratory viruses such as SARS-CoV-2 and influenza strains.

## SUPPLEMENTAL MATERIAL

Supplemental material aem.00774-25-s0001.docx Supplemental methods; Fig. S1 to S6.

Graphical abstract aem.00774-25-s0002.tif Visual depiction of study.

ASM does not own the copyrights to Supplemental Material that may be linked to, or accessed through, an article. The authors have granted ASM a non-exclusive, world-wide license to publish the Supplemental Material files. Please contact the corresponding author directly for reuse.
