# Supplementary material for: Evaluation of respiratory virus transmissibility and resilience from fomites: the case of 11 SARS-CoV-2 clinical isolates
Source: Appl Environ Microbiol. 2025 Aug 27;91(9):e00774-25. doi: 10.1128/aem.00774-25 (PMC12442357; doi:10.1128/aem.00774-25)
Supplement: Supplemental material — Supplemental methods; Fig. S1 to S6. [file aem.00774-25-s0001.docx]

**Supplementary materials**

**Summary**

**Material and Methods:** Cell lines; Virus isolation; Virus titration; Pseudovirus generation and titration; Pseudovirus mutation; Quantification of spike protein in pseudoviruses assay; XTT Assay for determination of cell viability and cytotoxicity; Immunofluorescence.

**Figure:** S1 Titration of pseudoviruses; S2 Bicinchoninic Acid Assay; S3 ELISA analysis; S4 Statistical analysis of inflammatory response; S5 Time-dependent activation of interferon; S6 Persistence mechanism of SARS-CoV-2 on copper**.**

**Materials and Methods**

**Cell lines**

Vero E6 (Vero C1008, clone E6; ATCC CRL-1586), HEK-293T ^CD81KO^ (Human, Epithelial Kidney cancer cell line, kindly offered Grove of the The Royal Free Hospital, University College London (DOI 10.1099/jgv.0.001512), MDCK (Madin Darby canine kidney, ATCC CCL-34) and 293T-ACE2/TMPRSS2 (Homo sapiens Embryonic Kidney Epithelial Cells Expressing Transmembrane Protease Serine 2 and Human Angiotensin-Converting Enzyme 2, BEI Resources, NIAID, NIH: NR-55293) were cultured in Dulbecco’s modified Eagle medium (DMEM) supplemented with non‐essential amino acids (NEAA), penicillin/streptomycin (P/S, 100 U/mL), HEPES buffer (10 mM), and 10% (v/v) heat-inactivated fetal bovine serum (FBS). 1 mg/mL of Geneticin (G418) was added to medium of Vero E6 that stably (Vero E6/TMPRSS2, NIBSC 100978).

Calu-3 (Human lung cancer cell line, ATCC HTB-55) was cultivated in Minimum Essential Medium (MEM) supplemented with NEAA (1×), P/S (100 U/ mL), 1 mM sodium pyruvate and 10% (v/v) heat-inactivated FBS. All cell lines were incubated at 37°C and 5% CO2 in a humidified atmosphere. All cell lines were regularly tested for mycoplasma (Lonza, LT07-218).

**Virus isolation**

1 mL of the transport medium of the nasopharyngeal swab (COPAN’s kit UTM® universal viral transport medium—COPAN) was mixed in a ratio of 1:1 with DMEM supplemented with P/S and Amphotericin B. The mixture was added to 80% confluent Vero E6 cells monolayer seeded in a 25 cm^2^ tissue culture flask. Adsorption occurs for 3h at 37°C, then 2 mL of DMEM supplemented with 2% (v/v) FBS and Amphotericin B was added to the monolayer. One day post-infection (dpi), the monolayer was washed in PBS and 3 mL of DMEM supplemented with 2% FBS and Amphotericin B were added. The cytopathic effect was monitored in inverted phase-contrast microscopy (Olympus CKX41) and the supernatant was collected at monolayer complete disruption (3 dpi), aliquoted and stored at -80°C (P1). A virus-specific PCR was performed on the virus stocks after a heat-inactivation at 56°C for 30. The viral genome was extracted using QIAamp Viral RNA Mini Kit (52906, Qiagen), following manufacturers’ instructions. The Superscript® III One-Step RT-PCR System (12574026, Invitrogen) with Platinum Taq DNA polymerase was used to perform both cDNA synthesis and PCR amplification in a single tube using RNA-dependent RNA Polymerase (RdRp) primers for SARS-CoV-2 (FW: 5’-CAAGTGGGGTAAGGCTAGACTTT–3’ and RE:5’–ACTTAGGATAATCCCAACCCAT–3’) and the following thermal cycling conditions: 50°C 20’, 50 cycle of 95°C 2’, 55°C 20’, 68°C 30’’ and 68°C 5’.

**Virus titration**

SARS-CoV-2 and influenza virus stocks were titrated using Endpoint Dilutions Assay (EDA, TCID_50_/mL). Either Vero E6, Vero/TMPRSS2, or MDCK cells were seeded into 96-well plates and infected at approximately 95% confluence with serial 10-fold dilutions of the virus stock. After 1 h of adsorption at 37 °C, the cell-free virus was removed, cells were washed with PBS, and complete medium supplemented with 2% FBS was added for Vero cells and DMEM no FBS supplemented with TPCK-trypsin (1:200) for MDCK cells. After 72 h, cells were observed in inverted phase-contrast microscopy (Olympus CKX41) to evaluate the presence of a cytopathic effect (CPE). TCID_50_/mL of viral stocks were then determined with the Reed–Muench formula.

**Pseudovirus generation and titration**

Lentiviral pseudoviruses were generated using the following spike proteins: G614D (NR-53765, BEI Resource), Beta strain (170449, Addgene), Delta strain (172320, Addgene), BA.1 (194602, Addgene), XBB.1.5 (196585, Addgene) and XBB.1, obtained by reverting the single mutation difference in XBB.1.5 using specific primers (doi: 10.3390/v12050513).

Briefly, HEK-293T ^CD81KO^ were seeded in a 75 cm^2^ tissue culture flask to be at 60-70% confluent the next day. 24 hours after cells were co-transfected with 5 plasmids from BEI Resource: 1.25 μg pHAGE with CMV-driven Luciferase-IRES-ZsGreen (NR-52516)

 0.275 μg pHDM with HIV Gag-Pol (NR-52517), 0.275 pHDM with HIV Tat (NR- 52518),  0.275 pRC with CMV-driven HIV Rev (NR-52519) and 0.425 μg pHDM- the previously S protein with C-term 21 bp. PEI (919012, Sigma-Aldrich) was mixed with DNA in a 1:3 ratio and incubated at room temperature for 30 minutes to allow complex formation prior to transfection. After 6 hours, the transfection medium was replaced with DMEM without FBS. At 48 hours post-transfection, the medium was collected and replaced, and the process was repeated at 72 hours post-transfection. Pseudovirus-containing media were then pooled and subjected to ultracentrifugation through a 20% sucrose cushion at 20,000 xg for 1 hour at 4°C using a Beckman 328 SW28 rotor. The pseudoviruses were aliquoted and stored at -80°C for further use.

Pseudovirus titer was determined by a luciferase assay. Vero/TMPRSS2 cells (3 x 10⁵ cells/mL) were seeded in a 96-well plate and incubated at 37°C for 24 hours. Serial 1:10 dilutions of pseudovirus were added to the cells, and the plate was spinoculated at 800 xg for 1 hour at 37°C for adsorption. Afterward, the pseudovirus-containing media were removed and replaced with fresh medium supplemented with 2% FBS.

72 hpi, cell supernatants were removed, and cells were lysed with 100 µL of Glo Lysis Buffer (E2661, Promega) for 15 minutes at room temperature with gentle agitation. Cell lysates were transferred to a luminometer plate, and 100 µL of Bright-Glo Assay Reagent (E2620, Promega) was added before measuring luminescence using a Victor3 luminometer (Perkin Elmer). For further assays, the dilution corresponding to Relative Luminescence Units (RLUs) greater than 1000-fold above background was selected.

**Pseudovirus mutation**

To investigate the role of the G446D mutation in the persistence mechanism, we used the plasmid encoding the spike protein of XBB.1.5 (196585, Addgene). Specific primers were designed to amplify the entire spike gene and revert the D446G mutation to the wild-type form (Table1). High-fidelity AccuPrime polymerase (12339016, Invitrogen) was used for amplification with the following thermal cycling conditions: 94°C 2 min, 35 cycle of 94°C 30 sec, 55°C 20 sec, 68°C 12 min and then 68°C 5 min. The amplification products were verified by agarose gel electrophoresis (1%), and the band corresponding to the full-length plasmid was excised. The plasmid was then digested with DpnI (R0176S, New England Biolabs) for 15 minutes at 37°C to remove methylated parental DNA. Electrocompetent *E. coli* (XL1-Blue; 200228, Agilent) cells were transformed and after 1 hour of recovery in SOC medium at 37°C with shaking, cells were plated on agar plates and incubated overnight at 37°C. Single colonies were picked, and plasmid DNA was extracted using the QIAprep Spin Miniprep Kit (27104, Qiagen).

The mutation was confirmed by Sanger sequencing (ABI 3100*,* SeqGen).

Once the mutation was verified, the wild-type XBB.1.5 (XBB.1.5_wt) was used to transfect the HEK-293T ^CD81KO^ as previously described. The pseudovirus titer was determined using the luciferase assay on Vero/TMPRSS2 cells and compared to the titer of XBB.1.5.

**Quantification of spike protein in pseudoviruses assay**

**BCA** Total protein concentrations were determined using the BCA Protein Assay Kit (A65453, Thermo Scientific, Pierce), following the manufacturer's instructions. Briefly, a standard curve was prepared using serial dilutions of bovine serum albumin (BSA) in PBS, with concentrations ranging from 0 to 2 mg/mL. For protein sample quantification, samples were diluted in PBS to fall within the linear range of the standard curve, using 10-20 µL per well. In a 96-well plate, 200 µL of BCA working reagent was added to each well and the plate was incubated at 37°C for 30 minutes. After incubation, the absorbance was measured at 562 nm using a microplate reader (Multiskan GO, Thermo Scientific). Protein concentrations (µg/mL) were determined by comparing the absorbance of each sample to the standard curve.

**ELISA** Briefly, 25 ng of each pseudovirus were coated onto 96-well plates and incubated overnight at 4°C. The plates were blocked with Protein-Free Blocking Buffer (37572, Thermo Fisher Scientific) to prevent non-specific binding, and incubated for 1 hour with SARS-CoV-2 spike antibody (anti-S rabbit 40150-R007, Sinobiological; diluted 1:1000). Subsequently, the plates were washed three times with PBS-T and a horseradish peroxidase (HRP)-conjugated anti-rabbit secondary antibody A24470, Invitrogen; diluted 1:5000) was then added for 45 minutes. The signal was developed by adding TMB (3,3',5,5'-Tetramethylbenzidine) substrate solution (N301, Thermo Fisher Scientific), and the reaction was stopped by adding sulfuric acid (410577000, Carlo Erba) to block the signal.

The absorbance was measured at 450 nm using a Multiskan GO plate reader (Thermo Scientific). The presence of spike variants was indicated by the level of absorbance, which was proportional to the amount of bound antibody, reflecting the pseudovirus spike protein interaction.

**XTT Assay for determination of cell viability and cytotoxicity**

Cell viability was assessed using the Cell Proliferation Kit II (XTT) (11465015001, Roche Diagnostics, Merck).

Vero/TMPRSS2 cells (3 x 10⁵ cells/mL) were seeded in 96-well plates and incubated for 24 hours. The culture medium was then replaced with medium containing different concentrations of the compounds Bafilomycin A1 (BFLA-1 1; SML1661, Merck), Camostat mesylate (SML0057, Merck), Tea tree oil, (TTO, W390208, Sigma-Aldrich), Daptomycin (DAP, SBR00014, Sigma-Aldrich) and Quercitin (QRE, PHR1488, Sigma-Aldrich) were incubated for 2 hours at 37°C. Following treatment, XTT reagent was added to each well, and the plates were incubated for 4 more hours. The optical density (OD) was measured at 450 nm (with a reference wavelength of 650 nm) using a Multiskan GO plate reader (Thermo Scientific).

**Immunofluorescence**

293T-ACE2/TMPRSS2 cells were seeded on glass coverslip for 1 day at 37°C and 5% CO2. Then, cells were fixed and permeabilized with ice-cold methanol-acetone (1:1) for 15 minutes at room temperature. After three washes with PBS, cells were incubated with the primary antibody (40150-MM08, Sino Biological; anti-S) for 1 hour at 37°C. Subsequentially, cells were washed three times with PBS and incubated with the secondary antibody labelled with Alexa fluorophore (A-11001, Thermo Fisher Scientific) for 30 minutes at 37°C. Cells nuclei were stained with Hoechst 33342 (94403, Sigma-Aldrich) for 15 minutes at 37°C. The images were acquired with Zeiss Axio Observer.Z1 microscope with QImaging Exi-Blue (Carl Zeiss, Oberkochen, Germany) at a 20-fold magnification. The obtained images were analyzed with ImageJ software (<https://imagej.nih.gov/ij/>).

**Figure**

**Figure S1**

**Figure S1.** **Titration of pseudoviruses expressing spike proteins from G614, Beta, Delta, BA.1, XBB.1, and XBB.1.5 variants.** Pseudoviruses were titrated using the Vero/TMPRSS2 permissive cell model and a series of ten 10-fold serial dilutions. After a 72hpi, relative light units (RLUs) were measured using a luciferase assay to quantify viral infectivity. The graph shows the mean and ± SD of RLUs signal of six replicate for each variant at each dilution level.

**Figure S2**

**Figure S2.** **Bicinchoninic Acid (BCA) Assay for Protein Quantification.** Total protein concentration was determined using the BCA assay. Psudovirus expressed the 6 different spike were incubated with BCA reagent, and absorbance was measured at 562 nm. A standard curve was generated using known concentrations of bovine serum albumin (BSA) to calculate the protein concentration in the samples (ug/ml).

**Figure S3**

**Figure S3.** **ELISA analysis for quantifying of spike proteins in pseudoviruses.** To evaluate whether spike protein expression on the pseudovirus surface is consistent across variants, rather than being variant-dependent we loaded an equal amounts (in ng) of protein of pseudoviruses produced in the permissive HEK293-ΔCD81 cell line. An antiS antibody was used to detect the protein across all variants. Despite a variation in signal intensity in G614D variants, which may be attributed to differences in the specificity of the primary antibody, an equal signal for the other VOCS was observed. Absorbance was measured at 450 nm. Data are shown as the mean ± SD, **** p < 0.0001.

**Figure S4**

**Figure S4. Statistical analysis of inflammatory response activation in the Calu-3 cell model.** The heatmap represented the statistical analyses (Two-way ANOVA) of the activation of RIG-1 and IFNB response at different time points (1, 3, 6, and 24 hours) following infection of 0.1(MOI). At 1hours, RIG-I was significantly upregulated in G614D compared to other VOCs (p < 0.0001), with no significant differences observed at 3 hours. At 6 hours, RIG-I mRNA levels were significantly higher in Delta compared to G614D (p < 0.001), and Delta showed a significant difference compared to Beta and XBB.1 (p < 0.1). At 24 hours, G614D exhibited a significant downregulation (p < 0.0001), while Beta showed differences compared to Delta and BA.1. For IFNB mRNA, at 1hours, G614D displayed significantly different levels compared to Beta, XBB.1, and XBB.1.5 (p < 0.0001). Beta was also significantly different from Delta and BA.1 (p < 0.0001), while Delta showed significant differences compared to XBB.1 and XBB.1.5 (p < 0.5). Furthermore, BA.1 was significantly different from XBB.1 (p < 0.5) and XBB.1.5 (p < 0.01). At 3 hours, IFNB levels were significantly different between G614D and Beta, XBB.1 (p < 0.0001), BA.1 (p < 0.5), and XBB.1.5 (p < 0.001). Delta also differed significantly from Beta (p < 0.5) and XBB.1 (p < 0.01). After 6 hours, IFNB mRNA levels were significantly different across all VOCs (p < 0.0001), except for comparisons between BA.1 vs Beta, XBB.1, and XBB.1.5; also, Beta vs XBB.1 and Delta vs XBB.1.5 showed no significant differences. Similar results were observed at 24 hours, with IFNB mRNA showing significant differences across VOCs, excluding the same pairwise comparisons as at 6 hours. Mean ± SD are shown, with statistical significance indicated by p-values.

**Figure S5**

**Figure S5.** **Time-dependent activation of interferon mRNA expression in response to VOCs in Calu-3 cells**. Graph showing mRNA expression of interferon genes after infection with 0.1 MOI, normalized to the GAPDH housekeeping gene and uninfected Calu-3 cells. Data represent the mean fold change across six independent replicates at each time point, highlighting how different viral variants activate the immune response in distinct ways. Statistical analysis (Two-way ANOVA) confirmed significant differences in immune response activation between variants and time points. A variable slope (four parameters) analysis was performed to fit the data, considering the differential expression levels. This analysis allows for a better understanding of the relationship between timepoint and the cellular response, accounting for varying levels of expression across conditions.

**Figure S6**

**Figure S6.** **Persistence mechanism of SARS-CoV-2 on copper** Fomite transmission was studied on copper at different time points. The persistence percentage was calculated based on the detected viral load (TCID_50_/ml) at each time point, relative to the titer of viral stock (TCID_50_/ml). The decrease in viral load was estimated by fitting a logistic growth curve. Data are shown as the mean ± SD, **** p < 0.0001.
