# Supplementary figures and images for: Evaluation of respiratory virus transmissibility and resilience from fomites: the case of 11 SARS-CoV-2 clinical isolates
Source: Appl Environ Microbiol. 2025 Aug 27;91(9):e00774-25. doi: 10.1128/aem.00774-25 (PMC12442357; doi:10.1128/aem.00774-25)

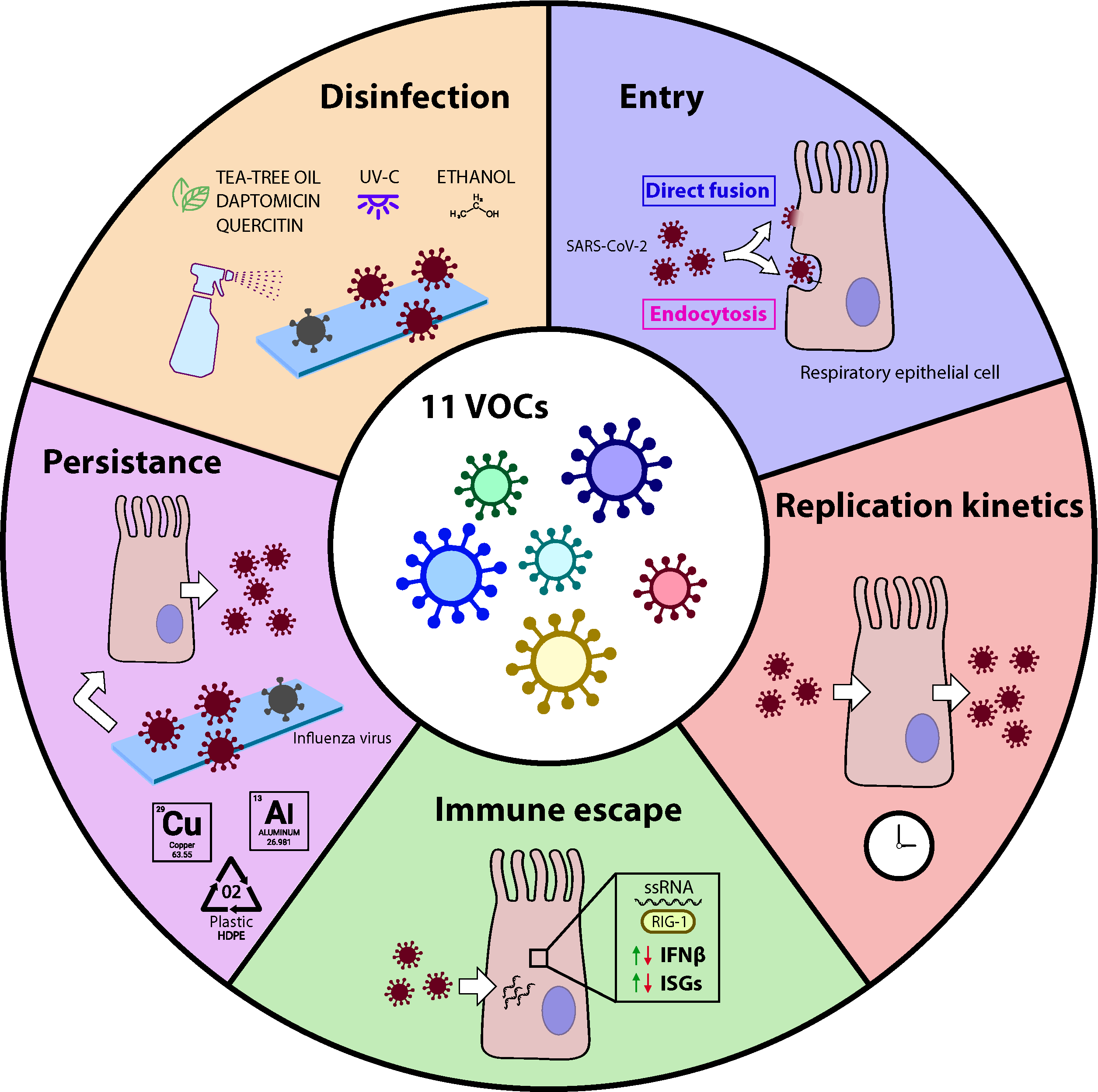

Supplement: Graphical abstract — Visual depiction of study. [file aem.00774-25-s0002.tif]
